# Supplementary material for: Inhibitory Mechanisms of Lekethromycin in Dog Liver Cytochrome P450 Enzymes Based on UPLC-MS/MS Cocktail Method
Source: Molecules. 2023 Oct 20;28(20):7193. doi: 10.3390/molecules28207193 (PMC10609143; doi:10.3390/molecules28207193)
Supplement: Supplementary file 1 [file molecules-28-07193-s001.zip › molecules-2625690-supplementary.pdf]

Supplementary Materials

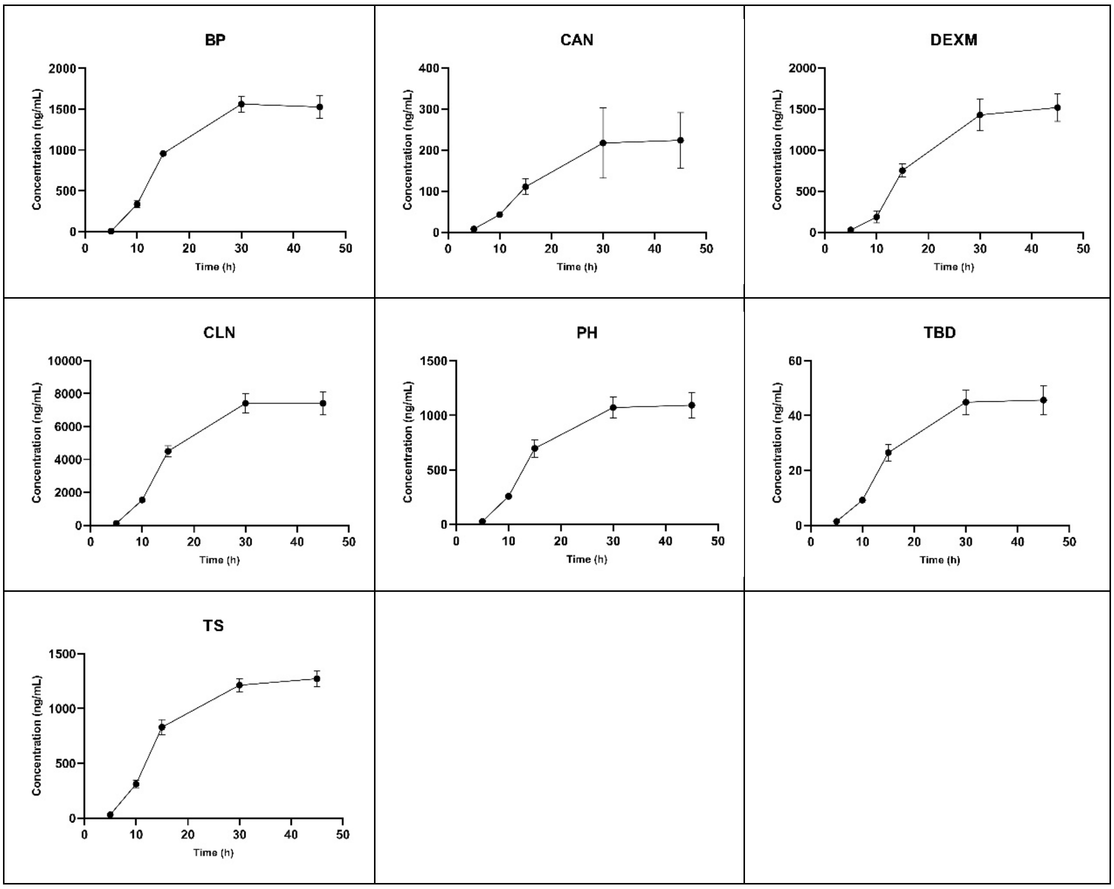

Figure S1. Optimized incubation time of bupropion (BP), coumarin (CAN), chlorzoxazone (CLN), dextromethorphan (DEXM), phenacetin (PH), tolbutamide (TBD), and testosterone (TS) in dog liver microsomes. Data are presented as mean±SD (n=3).

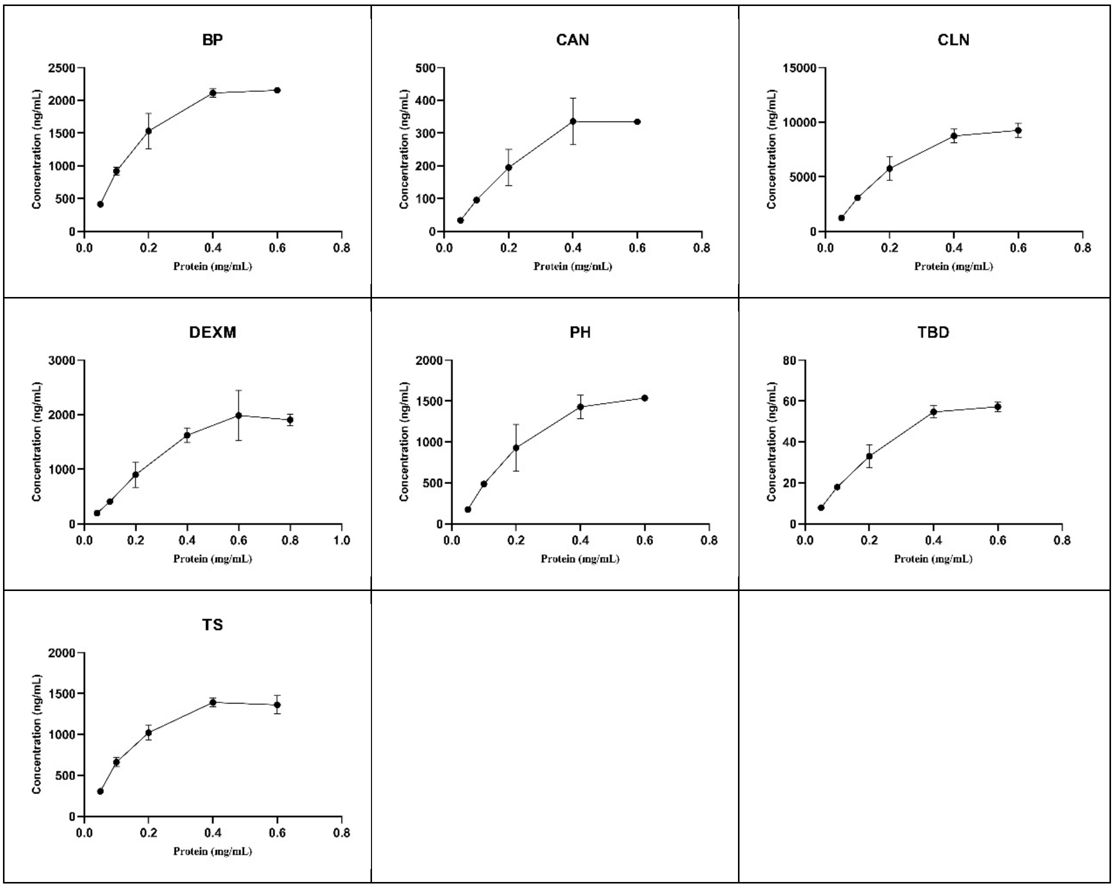

Figure S2. Optimized protein concentration of bupropion (BP), coumarin (CAN), chlorzoxazone (CLN), dextromethorphan (DEXM), phenacetin (PH), tolbutamide (TBD), and testosterone (TS) in dog liver microsomes. Data are presented as mean±SD (n=3).

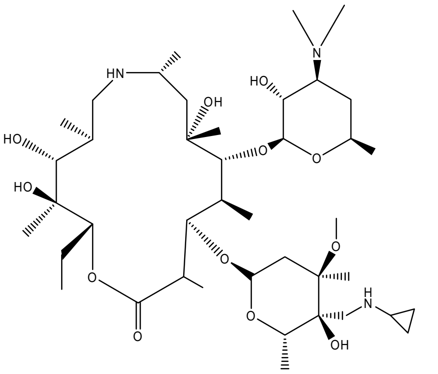

Figure S3. The structure of lekethromycin with molecule weight of 804.5 ($C_{41}H_{77}N_3O_{12}$)
